# Multi-Scale Modelling of Deformation and Fracture in a Biomimetic Apatite-Protein Composite: Molecular-Scale Processes Lead to Resilience at the μm-Scale

**DOI:** 10.1371/journal.pone.0157241

**Published:** 2016-06-14

**Authors:** Dirk Zahn, Patrick Duchstein

**Affiliations:** Lehrstuhl für Theoretische Chemie / Computer Chemie Centrum, Friedrich-Alexander Universität Erlangen-Nürnberg, Nägelsbachstraße 25, 91052 Erlangen, Germany; Harbin Institute of Technology, CHINA

## Abstract

Fracture mechanisms of an enamel-like hydroxyapatite-collagen composite model are elaborated by means of molecular and coarse-grained dynamics simulation. Using fully atomistic models, we uncover molecular-scale plastic deformation and fracture processes initiated at the organic-inorganic interface. Furthermore, coarse-grained models are developed to investigate fracture patterns at the μm-scale. At the meso-scale, micro-fractures are shown to reduce local stress and thus prevent material failure after loading beyond the elastic limit. On the basis of our multi-scale simulation approach, we provide a molecular scale rationalization of this phenomenon, which seems key to the resilience of hierarchical biominerals, including teeth and bone.

## Introduction

Teeth and bones are sophisticated inorganic-organic composite materials of exceptional mechanical properties [1,2]. Hardness is provided by the inorganic component, like calcium phosphates or iron oxides. On the other hand, fracture resilience of such biominerals arises from the organic part, e.g. amelogenin and collagen proteins [1,2,3,4]. While we are still at the beginning of understanding the molecular scale mechanisms involved, a two-fold concept for rationalizing the mechanical properties of biominerals evolved within the past decade. i) At the interface of the two rather dissimilar constituents, the materials properties change from hard/brittle to soft/elastic, thus providing dedicated regions of particular properties. ii) Moreover, biominerals used as teeth or bone exhibit hierarchical structures which lead to well-defined arrays that determine the location of harder and softer domains [2].

While processes at the organic-inorganic interface are confined to the nanometer scale, the structural hierarchy of teeth and bone directs deformation and fracture at the 10 nm to micrometer scale. As a consequence, the rationalization of mechanics must span length scales from atoms to micrometers. This poses considerable challenges to both experiment and theory [5]. The mechanisms operating at different length scales are closely linked, and characterization of meso and nano scale processes requires insights from the molecular scale.

In the present study, we employ recently developed molecular models to create a coarse-grained model that accounts for deformation and fracture up to the micrometer scale. For this purpose, detailed knowledge of the structural hierarchy is required—which is not yet available for biogenic minerals. This motivated the choice of the well-defined biomimetic apatite-collagen composite developed by Kniep and coworkers for our simulations [5,6,7,8]. The model is biomimetic to human enamel, but exhibits a simpler and more ordered structural hierarchy that could recently be resolved with an extensive series of experiments and molecular simulations [6]. While enamel contains the structurally much less defined protein amelogenin, it was shown that the collagen-based composite of Kniep and coworkers exhibits a comparable nano-mosaic structure [7,8]. Moreover, previous simulation studies on this compound revealed enamel-like mechanisms of elastic/plastic deformation upon compression [9,10] and shearing of rods upon indentation [11]. A yet missing link to enamel—and hence crucial support for suggesting apatite-collagen composites as a new generation of dental repair materials—is comparability in terms of fracture mechanisms. The aim of the present work is thus the creation of sufficiently large model systems to study meso-scale fracture patterns.

## Models and Methods

### Atomistic Simulations

The creation of a molecular model of apatite-collagen composites was subject of a series of theoretical studies ranging from “brute-force” ion association at over-concentration [12], diffusion-controlled ion association and composite nucleation [8] to incorporation of apatite crystallites into collagen matrices [10,13]. In the present work, the atomistic simulations are based on a recently developed molecular model [10] of a single collagen triple-helix embedded in single-crystalline apatite. This is transferred into a larger super-cell, comprising a hexagonal pattern of 4 explicit collagen proteins embedded into 10×10×10 unit cells of apatite [9]. Accordingly, our model was created on the basis of combined experimental and modelling work to best mimic the composite material as synthesized by Kniep and coworkers [6,7,8,9,10].

Periodic boundary conditions are applied to mimic a bulk material. Molecular dynamics simulations using a time step of 1 fs and Ewald summation with a real space cut-off of 12 Å are employed to first relax the model at constant pressure and constant temperature, imposing 1 atm and 300 K, respectively. The interaction potentials are adopted from earlier work reported in [10], which so far was successfully employed for studying ion association [8], composite formation [10], deformation [9] and indentation [11].

The simulation cell is then fixed along the b and c directions, whilst the system is subjected to stretching along the a-axis. This is implemented as molecular dynamics simulations using constant scaling rates to allow for unbiased system relaxation to non-elastic deformation and fracture pathways. During this process, a thermostat is used to maintain 300 K and thus damp the heat resulting from exerting mechanical work. A variety of strain rates are tested to ensure convergence of the deformation and fracture mechanisms which turn out to be strain rates of 1 m/s or less. For the production runs, we use a stretching rate of 0.25 m/s. This is well below the critical rate of 1 m/s observed for the simulation model and also appears to be a reasonable estimate for the speed of chewing and biting.

### Coarse-grained Simulations

**Fig 1:**
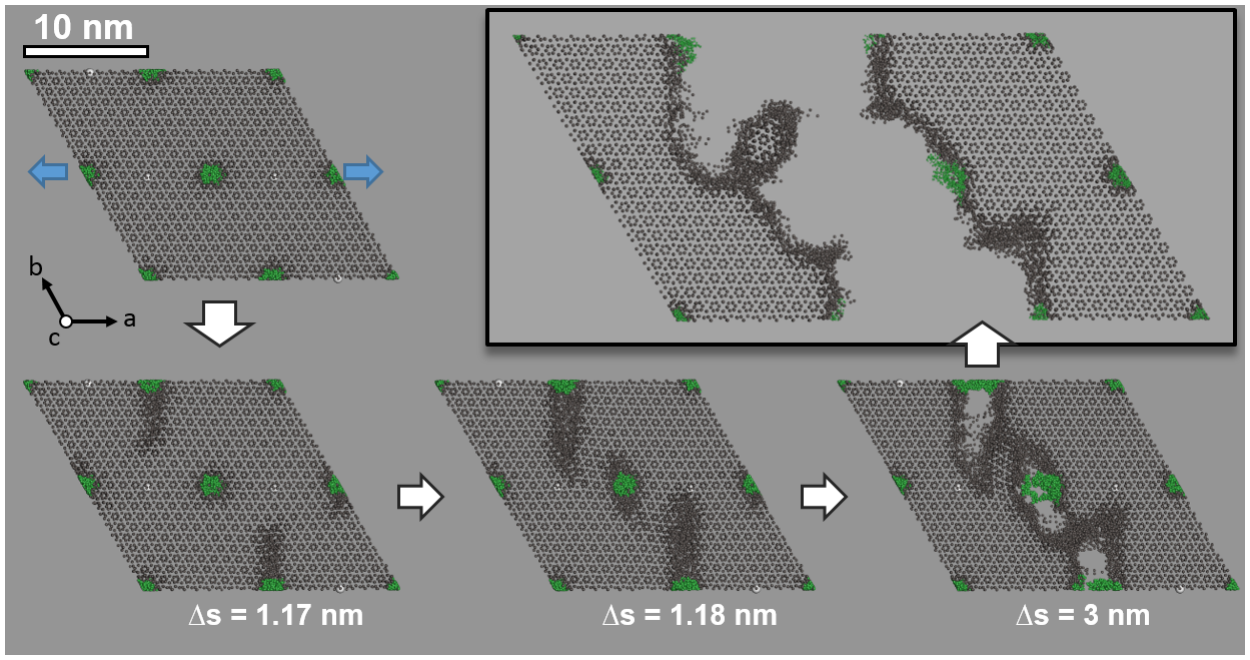
Atomistic simulation model mimicking the composite material at the nanometer length scale. While the overall model comprises 212.340 atoms, only calcium ions (grey) and the backbone of the collagen triple-helices (green) are shown. Using 3-dimensional periodic boundary conditions, the system is stretched (blue arrows) along the a-axis to induce fracture (which occurs at Δs ≈ 3.5 nm). Plastic deformation preferentially occurs at the organic-inorganic interface and gives rise to rough fracture patterns that are determined by the arrangement of the proteins within the composite.

**Fig 2:**
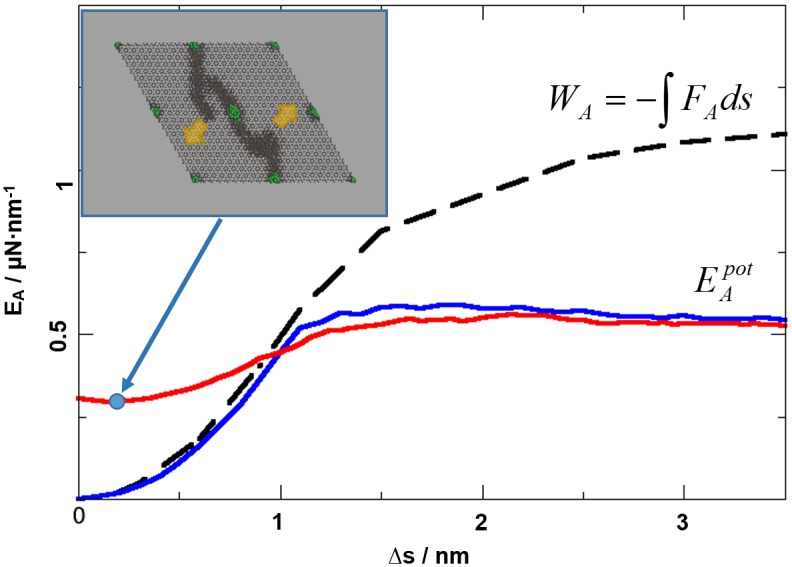
Potential energy (blue curve) and mechanical work (black curve) per nm^2^ as functions of model stretching Δs along the (100) direction. Upon elongation by about 1 nm, the deformation mechanism changes from elastic to plastic. After stretching up to the fracture limit of 3.5 nm, relaxation is investigated by reversing the strain rate. The inset at the top left shows that the system does not immediately recover to the pristine configuration, but retains considerable plastic deformation. As a consequence, the corresponding potential energy profile (red curve) exhibits an offset in both energy and equilibrium geometry. Note that despite the limited dimensions of the atomistic model, the onset of fracture bifurcation (highlighted by yellow arrows) is—at least qualitatively—already observed.

**Fig 3:**
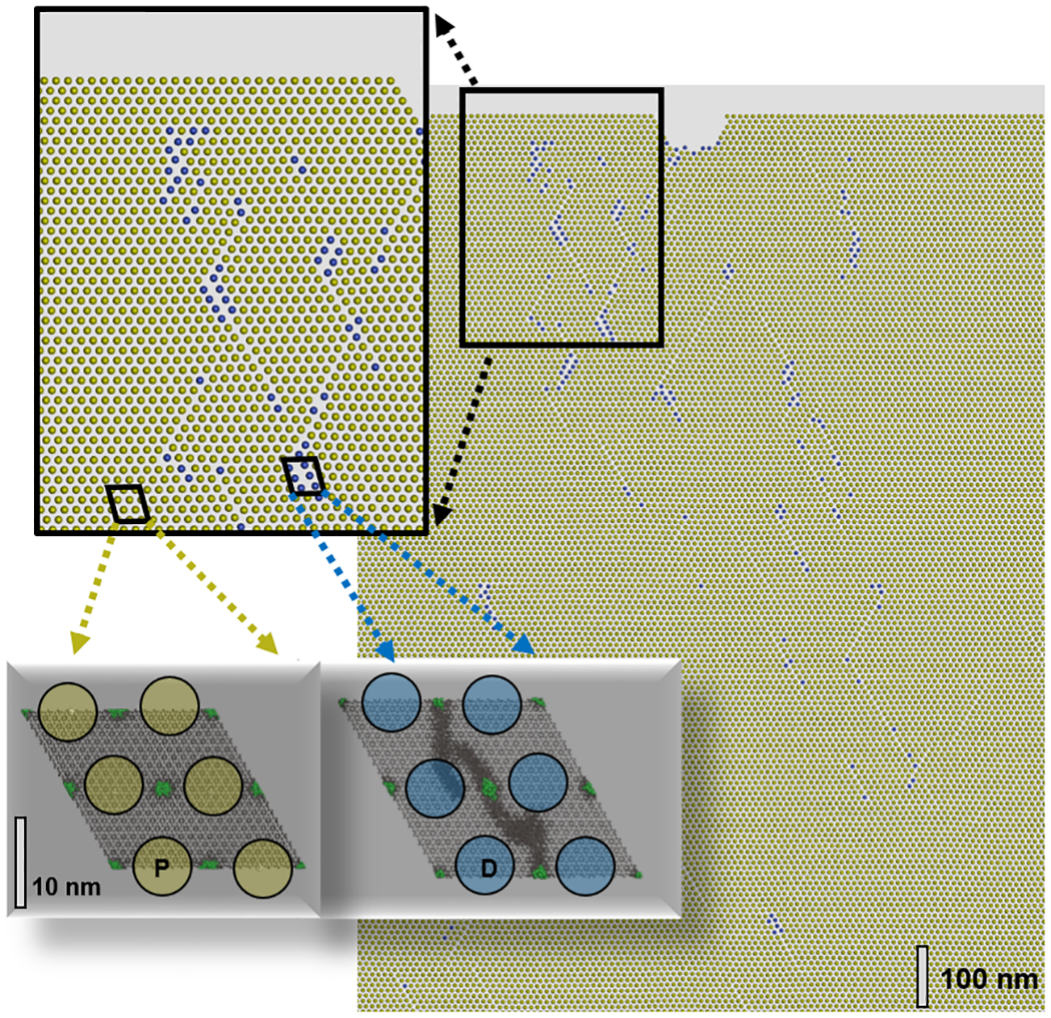
Coarse grained model mimicking the composite material at the μm scale. Right: scale-up model comprising 90.000 building blocks. The system was loaded by implementing 5% elastic deformation along the horizontal direction. The snapshot was taken 100.000 time-steps after allowing the system to relax. The inset at the lower left illustrates the coarsening of the atomistic model in terms of two only types of building blocks, namely pristine (P) and damaged (D) building blocks, shown in yellow and blue, respectively.

The coarse-grained setup is chosen as a particle-based model that reduces the previously described atomistic resolution to a minimalistic level of detail, whilst mimicking the elastic, plastic and fracture behavior of the composite. Along this line, the simulation system is divided into hexagonal prisms used as building blocks that each account for a collagen triple-helix and 10×10×10 unit cells of apatite. The position of the individual prisms is only considered within the xy plane, thus leading to a 2-dimensional model (which implicitly models periodic boundary conditions along the c axis). The coarse-grained model was divided into two species of building blocks to mimic i) the pristine composite, as observed before plastic deformation, and ii) the damaged composite, as observed from relaxation after loading beyond the elastic limit (figs 1 and 2). See also fig 3 for an illustration of the coarse-grained model and its relation to the atomistic structure.

To determine the interaction potentials of the building blocks, the coarse-grained model is subjected to deformation and fracture processes analogous to the atomistic model. The building-block species i) is chosen to reproduce the potential energy profile of the pristine (P) composite as a function of increasing strain along the a-axis. This was achieved by a harmonic interaction potential (mimicking the elastic deformation regime) that *is truncated at a critical distance delimiter to account for fracture*. Moreover, the energy profile of relaxing the atomistic model after exposure to plastic deformation is employed to parameterize the interactions of the building-block species ii) mimicking ‘damaged’ (D) domains of the composite (see also fig 3, highlighting pristine and damaged building blocks in yellow and blue, respectively). Similar to the atomistic model, the corresponding force-field is described by a truncated harmonic potential, however using softer spring constants and larger equilibrium distances of the building-blocks. The interaction models resulting from the energy profiles shown in fig 2 read:

$$\begin{array}{l} i)\;V_{P}\left(r\right)=\{\begin{array}{c} 1.77\cdot 10^{5}kJmol^{-1}\cdot \left[{\left(r-r_{o}^{P}\right)}^{2}-{\left(r_{c}-r_{o}^{P}\right)}^{2}\right]\;r\leq r_{C}\\ 0\;r>r_{c}^{P} \end{array}\\ ii)\;V_{D}\left(r\right)=\{\begin{array}{c} 1.10\cdot 10^{5}kJmol^{-1}\cdot \left[{\left(r-r_{o}^{D}\right)}^{2}-{\left(r_{c}-r_{o}^{D}\right)}^{2}\right]\;r\leq r_{C}\\ 0\;r>r_{c}^{D} \end{array} \end{array}$$

With *r* being the center-of-mass to center-of-mass distance of the building blocks, and *r*_*o*_^*P*^ = 9.17 nm and *r*_*o*_^*D*^ = 9.35 nm being the size of the building blocks mimicking the relaxed pristine and damaged models, respectively. The cut-off threshold for fracture was found as *r*_*c*_^*P*^ = 9.65 nm and *r*_*c*_^*D*^ = 9.82 nm for the pristine and damaged building-block species, respectively. The potential type i) is only used for building-blocks within an entirely undamaged (i.e. pristine composite) nearest neighborhood. Switching from pristine→damaged building blocks is imposed by the separation of at least one neighbor by *r*_*c*_^*P*^ = 9.65 nm (see also fig 3). The reverse process, i.e. self-healing, requires dramatically longer time-scales compared to damaging from plastic deformation and is neglected in this simple, coarse-grained model.

To demonstrate μm scale deformation and fracture, a coarse-grained model of 300×300 building-blocks was subjected to 5% elastic deformation, followed by relaxation analysis from particle dynamics simulations at constant strain. As a consequence of considerable model reduction, this relaxation process of building-blocks rearrangement may be explored from molecular dynamics simulations using a time-step of Δt = 350 fs. This allows assessing drastically larger scales in both length and time within reasonable computational efforts.

## Results and Discussion

While synthesized from calcium phosphate, precipitation in disordered gelatin gels, the enamel-like apatite-collagen composites as elaborated by Kniep and coworkers exhibit a remarkable degree of ordering [6,7]. At the molecular scale, the incorporation and orientation of apatite motifs within the collagen triple-helix leads to an alignment of the c axis of apatite with the collagen fibers [8]. Within the ab plane, the collagen triple-helices are arranged as a hexagonal pattern, giving rise to a hierarchical composite structure [7,8]. This ordered pattern of single-crystalline apatite domains and collagen-apatite interface zones was recently shown to account for the localization of plastic deformation during compression and indentation along the c-axis [9,11].

To explore fracture processes at the molecular scale, the same atomistic model as in the previously described studies is employed [9]. We however impose expansion along the a axis by linear scaling of both atomic coordinates and the corresponding cell vector. While this in principle would reflect an ideal elastic deformation, molecular dynamics simulations are performed to analyze the relaxation process. This relaxation process encompasses several regimes, i.e. elastic deformation, plastic deformation, the nucleation of cavities and fracture (fig 1). Extensive molecular dynamics simulations were carried out to ensure sufficiently slow strain rates (25 cm/s, cf. simulation details) and to allow for the discrimination of the individual mechanistic steps. On this basis, the transition from elastic to plastic deformation is identified at the organic-inorganic interface of the composite. While initially involving all proteins, at later stages we find nearby domains of plastic deformation to merge, hence leading to increasingly continuous zones of ‘damaged’ apatite (fig 1). At the same pace, stress is reduced and the rest of the simulation system relaxes. This is reflected in the stress-strain profile as a gradual reduction of the force opposing further pulling and roughly constant potential energy (fig 2). From a molecular perspective, relaxation also involves the ‘self-healing’ of damaged regions near collagen molecules that are not involved in the fracture line (fig 1, lower right). This observation is fully in line with our earlier analysis of self-healing after moderately compressing beyond the elastic limit [9].

The fracture line illustrated in fig 1 originates from coalescing cavities, each nucleated at the organic-inorganic interface. Hence, an immediate implication of this feature is that fracture lines tend to follow curves that connect the proteins. Instead of sharp cuts along a crystallographic plane (as characteristic for brittle compounds, including non-collagenous apatite) we thus find that fracture pathways are determined by the arrangement of the organic component within the composite. The hierarchical nature of the composite, however, implies a hexagonal pattern of proteins and thus zig-zag type fracture lines (fig 1, top right). As a consequence, a considerable portion of the mechanical energy applied during pulling is transferred into particularly rough surfaces, hence increasing fracture energy beyond the level of pure apatite.

A further mechanism of increasing fracture resilience is given by the chance of fracture line bifurcation. Indeed, the onset of fracture curve branching may be seen for the atomistic model shown in fig 2, lower right. The 20 nm sized simulation model is however too small to allow the explicit analysis of branched fracture patterns. This motivated the development of a simplified, coarse-grained model. The underlying building blocks (as described in detail in the methods section) account for the elastic deformation and rupture (fig 2, blue curve) of the pristine composite model (P) and also consider zones of plastic deformation by mimicking the stress-strain diagram as observed for relaxation of a damaged (D) composite model (fig 2, red curve). An illustration of the coarse-grained model is given in fig 3.

**Fig 4:**
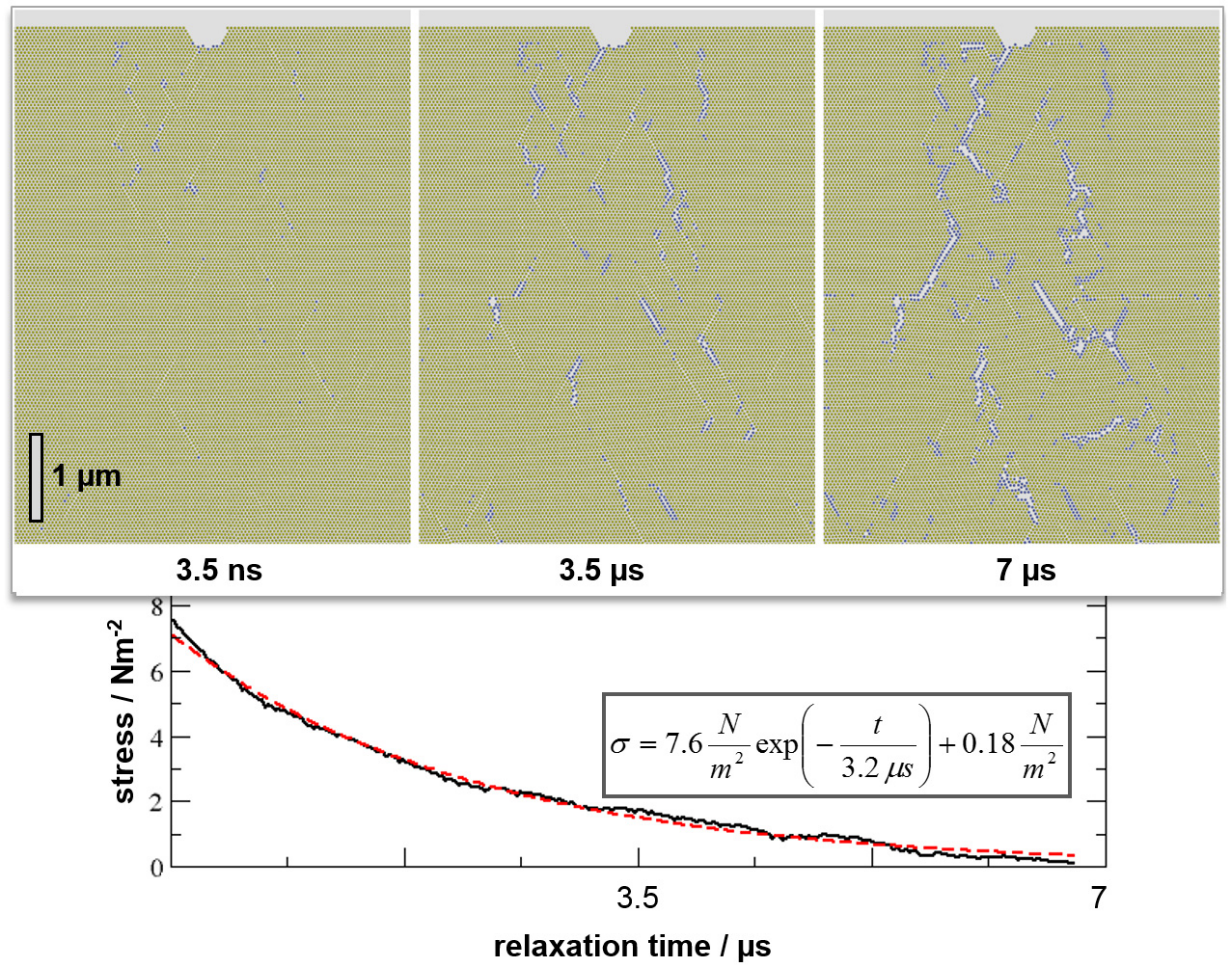
Relaxation of the scale-up model after loading and holding 5% strain along the horizontal direction. The initially elastically deformed model evolves in favor of a heterogeneous fracture/plastic deformation pattern and exponential decay of the stress profile is observed (fitted curve shown in red). Note that instead of a single fracture line (which would imply material failure) we observe a non-percolating pattern of a large number of micro-fractures.

To account for a setup commonly used in loading experiments, we spontaneously applied strain and then explored relaxation whilst maintaining constant strain. Moreover, the model of 90.000 hexagonally arranged building-blocks is subjected to a kink at the upper surface to explore possible crack initiation and propagation from a pre-existing imperfection of the material. Fig 3 shows the corresponding μm scale model after application of 5% strain along the horizontal direction. Though initially implemented as elastic deformation, the model system quickly relaxes in favor of plastic deformation and fracture. The snapshot in fig 3 is taken at the very beginning of this relaxation process. Nevertheless, the onset of fracture pattern formation in terms of a large number of micro-fissions, embedded by damaged composite zones may be observed. During further relaxation, the number of cracks increases and the stress decays exponentially (fig 4). Despite offering a kink as crack initiator, we still find that crack propagation barely exceeds the 100 nm scale and instead observe crack termination by fracture line bifurcations (fig 4, top right). During 7 μs simulation time, stress is reduced by an order of magnitude in the course of the formation of a branched pattern of micro-cracks. This observation is very similar to the micro-cracks found in enamel after indentation experiments [14,15,16]. For both composites, we suggest that the underlying structural hierarchy of how harder (pure apatite) and softer (apatite-protein interfaces) zones are distributed in the composites accounts for the disperse nature of fissions which hinders rupturing (requiring a continuous fracture line).

## Conclusions

The biomimetic composite investigated in this work represents a synthetic material, and thus a simplification of biogenic apatite-protein composites such as teeth and bone. The number of structural and mechanical similarities is however appealing. From a viewpoint of fundamental research, the discussed apatite-collagen composite serves as a well-defined model (including reproducible synthesis!) whose structure has been characterized to a level of detail that is barely matched [6]. In parallel to experimental efforts, molecular simulations spanning from the early stages of composite nucleation to the characterization of the bulk composite structure offer molecular scale insights that explain the peculiar interplay of organic and inorganic constituents [8,10]. Moreover, the resulting mechanical properties such as interface-driven plastic deformation and slip zone formation could be elucidated at the atomic level of detail [9,11]. This profound understanding is key to model reduction, namely coarsening into building blocks—which still convey the peculiar mechanical properties of the composite. The observed patterns of branched micro-cracks leading to exponential stress reduction show striking similarities to enamel. This suggests that the link between molecular/nanoscale structure and μm-scale fracture made for the apatite-collagen model should be transferrable to biogenic composites at least from a qualitative viewpoint.

A more technical implication is that the biomimetic composite is not only chemically similar to enamel, but also shows a similar hierarchy of mechanisms accounting for the mechanical properties. In other terms, our simulations suggest that the biomimetic composite exhibits fracture resilience very similar to enamel. In combination with the bio-compatibilty of the biomimetic apatite-collagen composite and its affinity to enamel/dentin (both originating from chemical similarity) this provides further evidence for an excellent potential as a dental filling material.
